# Mechanisms of hemoglobin cycling in anemia patients treated with erythropoiesis-stimulating agents

**DOI:** 10.1371/journal.pcbi.1010850

**Published:** 2023-01-24

**Authors:** David J. Jörg, Doris H. Fuertinger, Peter Kotanko

**Affiliations:** 1 Computational Medicine Group, Global Medical Office, Fresenius Medical Care Germany, Bad Homburg, Germany; 2 Renal Research Institute, New York, New York, United States of America; 3 Icahn School of Medicine at Mount Sinai, New York, New York, United States of America; University at Buffalo - The State University of New York, UNITED STATES

## Abstract

Patients with renal anemia are frequently treated with erythropoiesis-stimulating agents (ESAs), which are dynamically dosed in order to stabilize blood hemoglobin levels within a specified target range. During typical ESA treatments, a fraction of patients experience hemoglobin ‘cycling’ periods during which hemoglobin levels periodically over- and undershoot the target range. Here we report a specific mechanism of hemoglobin cycling, whereby cycles emerge from the patient’s delayed physiological response to ESAs and concurrent ESA dose adjustments. We introduce a minimal theoretical model that can explain dynamic hallmarks of observed hemoglobin cycling events in clinical time series and elucidates how physiological factors (such as red blood cell lifespan and ESA responsiveness) and treatment-related factors (such as dosing schemes) affect cycling. These results show that in general, hemoglobin cycling cannot be attributed to patient physiology or ESA treatment alone but emerges through an interplay of both, with consequences for the design of ESA treatment strategies.

## Author summary

Patients with kidney failure often suffer from anemia, a disease characterized by a shortage of red blood cells. In clinical practice, this shortage is assessed through measurements of blood hemoglobin concentration. Anemia is typically treated using drugs that mimic erythropoietin, the body’s own hormone regulating red blood cell generation in healthy subjects. During anemia treatment, a widespread phenomenon termed “hemoglobin cycling” may emerge, whereby blood hemoglobin levels oscillate with high amplitude around a target concentration which the therapy aims to achieve. This is an undesired condition, which also necessitates frequent adjustment of the drug dose. The causes of hemoglobin cycling are not understood on a fundamental level. We developed a simple mathematical model that describes physiological and treatment-related effects. In this model, cycling emerges because of long delays between drug administration and their effect on hemoglobin concentration and vice versa, leading to a negative-feedback loop that reinforces oscillations. We use our model to develop statistical indicators of this type of hemoglobin cycling in clinical data and analyze how physiological and treatment-related factors may contribute to the appearance of cycling. These results can help to understand how to modify existing treatment schemes to avoid hemoglobin cycling in the first place.

## Introduction

**Fig 1:**
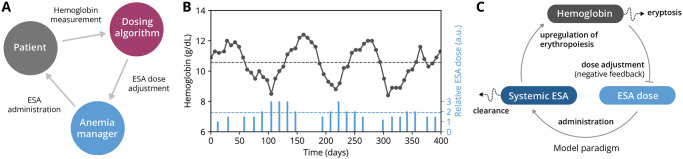
General features of ESA therapy and hemoglobin cycling. (A) Simplified representation of the treatment loop involved in erythropoiesis-stimulating agent (ESA) therapy: An anemic patient’s hemoglobin levels are measured on a regular basis and inform ESA dose adjustments via a dosing algorithm and/or an anemia manager’s decision. The given ESA dose, in turn, affects the patient’s hemoglobin levels. (B) Historical record of hemoglobin (Hb) cycling in an anemic hemodialysis patient showing Hb measurements (dots) and ESA administrations (bars). Dashed lines indicate mean values for the respective quantities on the shown time horizon. (C) Schematic depiction of the biomedical model with three variables (Hb levels, current ESA dose prescription and systemic ESA levels) capturing the key features of the feedbacks shown in panel A. The model is described in detail in the Methods section.

Homeostasis of red blood cells (RBCs) is a vital process regulated by a cross-talk between the bone marrow and the kidneys. Depending on the instantaneous arterial blood oxygen content, healthy kidneys adjust their release of erythropoietin (EPO), the major regulator of RBC generation in the bone marrow [1]. In patients with chronic kidney disease (CKD), this homeostatic feedback is compromised, often entailing anemia. Anemia results in impaired oxygen delivery to tissues and organs, giving rise to a host of clinical manifestations, such as fatigue, cognitive impairment, tachycardia and, in extreme cases, organ failure [2]. Treatment of anemia aims at replacing the endogenous EPO feedback through recurrent administrations of erythropoiesis-stimulating agents (ESAs) such as recombinant human erythropoietin (rHuEPO). Similar to endogenous EPO, ESAs downregulate both apoptosis of erythroid progenitors and the selective destruction of young RBCs (neocytolysis) [1, 3, 4], eliciting a response that takes effect on multiple time scales from minutes to weeks. This delayed physiological response, together with a large variability in patients’ ESA responsiveness and RBC lifespan, makes ESA dosing notoriously challenging. Frequent dose readjustments are needed to achieve and maintain a patient’s blood hemoglobin (Hb) concentration at a specified target level, especially after severe perturbations such as bleeding episodes. Effectively, this procedure of recurrent Hb measurements, ESA dose readjustment and administration mimics (and aims to replace) the kidney’s failing ability to adequately regulate and release endogenous EPO based on blood oxygen content (Fig 1A). In many clinical settings, this is achieved by treating entire populations of anemic kidney patients using a one-size-fits-all ESA treatment scheme or algorithm, often represented as a decision tree, in which dosing decisions are based on threshold criteria involving most recent Hb measurements [5, 6].

A common problem in anemia treatment is the emergence of ‘hemoglobin cycling’ patterns, whereby the Hb concentration periodically over- and undershoots a target value or range [5–9] (Fig 1B). Hb cycles can vary in duration from several weeks to months and entail large variations in oxygen supply to vital organs [5]. Hence, Hb cycling and, more generally, Hb variability are associated with comorbidities and poor clinical outcomes. In patients with end-stage kidney disease (ESKD), an increase of 1 g/dL in Hb standard deviation has been reported to be associated with a 33% increase in rate of death [10], although the causal role of Hb variability has been debated [11]. High-amplitude Hb fluctuations around a target range have also been associated with a higher risk for cardiovascular disease [12].

The general causes of Hb cycling are likely multifactorial and still under debate. The emergence of Hb cycling has been associated with patient variability in ESA responsiveness, inadequate Hb target ranges and ESA dosing practices relying on Hb point measurements; as well as changes in fluid, iron and nutritional status but also external events such as bleedings, illnesses leading to inflammation and hospitalizations [5, 6, 9]. Pharmacologically, the problem of cycling has been tried to be mitigated through the development of longer-acting drugs such as continuous EPO receptor activators (CERAs) [13]. On the level of treatment practices, in order to overall improve anemia management, approaches from mathematical modeling, control theory and machine learning aim to capture and predict a patient’s response to ESAs and inform general treatment algorithms or personalized dosing decisions [14–25]. While such approaches have successfully improved therapy, their complex mathematical and procedural structure makes it difficult to isolate and identify the physiological and therapy-related factors that are specifically related to Hb cycling. Insights into specific mechanisms of cycling and the dynamical imprints that they leave on clinical data remain rare.

A sensible candidate for a cycle-inducing mechanism is the effective feedback mediated by ESA therapy, in which dosing decisions are based on Hb levels and Hb levels in turn depend on previous dosing decisions. It has been noted that ESA treatment protocols may elicit a too strong and too delayed ESA dose change following the departure from the Hb target range, thereby systematically causing Hb overshoots and undershoots [5, 25]. From a systems-level point of view, ESA treatment establishes a ‘homeostat’ for the blood Hb concentration, i.e., a delayed negative-feedback loop between the patient’s physiological response and ESA dosing decisions (Fig 1C). While being designed to replace the homeostatic feedback of the kidney, the presence of long (physiological and treatment-related) delays in this feedback loop suggests it may constitute a systematic driver of Hb cycling. Here we term this mechanism of Hb cycling ‘self-sustained’ (in the sense of the closed feedback shown in Fig 1A) and we investigate self-sustained Hb cycling both theoretically and through clinical data analysis. We establish a minimal theoretical model of the corresponding feedback mechanism, which enables us to extrapolate the physiological and treatment-related features contributing to self-sustained Hb cycling. Furthermore, we use the model to identify dynamical hallmarks of self-sustained Hb cycling and establish the presence of such hallmarks in historic clinical datasets. The implications of these findings for the analysis of existing and future ESA treatments are discussed.

## Results

### Biomedical modeling scheme

To study the feedback mechanism that leads to self-sustained Hb cycling, here we introduce a paradigmatic mathematical model that captures the core features of generic ESA administration schemes and a patient’s physiological response. The model describes the dynamics of three variables: (i) the Hb levels of an anemic patient who is subject to an ESA treatment scheme, (ii) the prescribed ESA dose and (iii) the bioavailable amount of ESA within the patient’s body (Fig 1C; see Methods for a formal description of the model). Importantly, these three components form a negative feedback loop, with higher Hb levels leading to smaller ESA doses, whereas higher ESA doses lead to higher Hb levels. While this modeling scheme relies on simplifications as compared to more realistic computational approaches, it contains the important dynamical elements to illustrate the dominant mechanism underlying self-sustained Hb cycling and is able to produce testable results.

**Fig 2:**
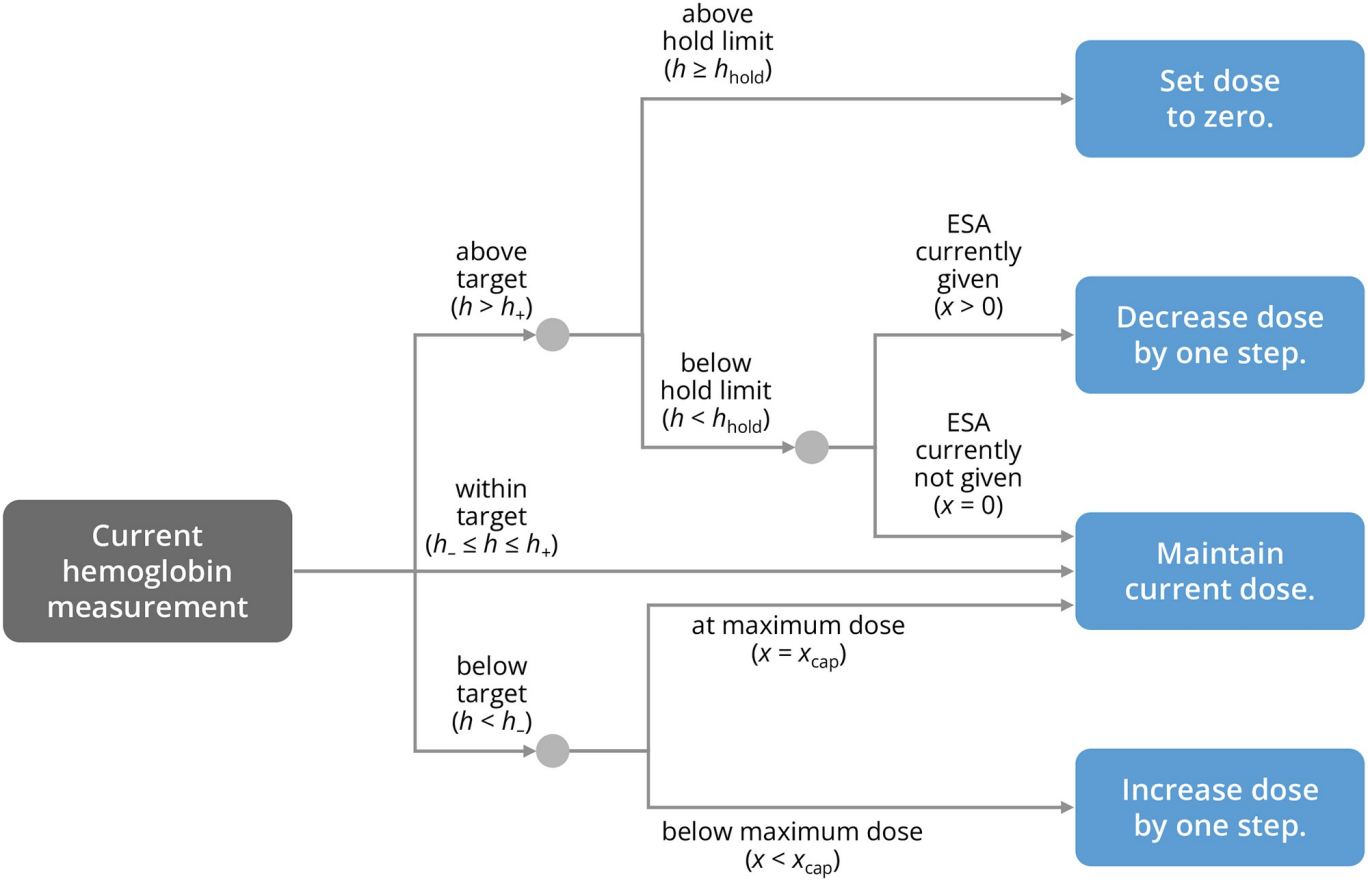
Simplified ESA dosing scheme. Decision tree used in the biomedical model that represents the prototypical features ESA dosing schemes based on a patient’s current hemoglobin measurement.

In this model, decisions to administer ESA are made on a biweekly basis and follow a simple set of rules mimicking an anemia manager’s or a treatment algorithm’s decision pattern based on measured patient Hb levels (Fig 2): If the patient’s Hb is located within a defined target window (which we here set to 10–11 g/dL), the previously administered ESA dose is maintained. If the patient’s Hb level is below/above the target window, the ESA dose is increased/decreased in discrete steps but cannot exceed a specified maximum dose. If the patient’s Hb level exceeds a certain critical threshold (the ‘hold limit’), ESA administration is entirely suspended until the patient’s Hb level drops below the target window. After an ESA dose is determined, ESA is injected, captured by a jump in the systemic ESA levels which subsequently start to decay with a characteristic half-life. The patient’s Hb levels respond to an administered ESA dose in a nonlinear fashion, with small ESA concentrations having little to no effect while large concentrations lead to a saturating effect. In addition, the Hb response occurs with a time delay w.r.t. ESA administration; since ESA prevents apoptosis of erythropoietic progenitors, this delay represents an effective average until these multiple mechanisms of action take effect on Hb levels [26]. The model also accounts for biological fluctuations in the Hb dynamics as well as for Hb measurement errors which may affect the decision to alter the ESA dose. In summary, the model describes a closed feedback loop, within which the patient’s Hb levels depend on the ESA dose and vice versa (Fig 1C).

**Fig 3:**
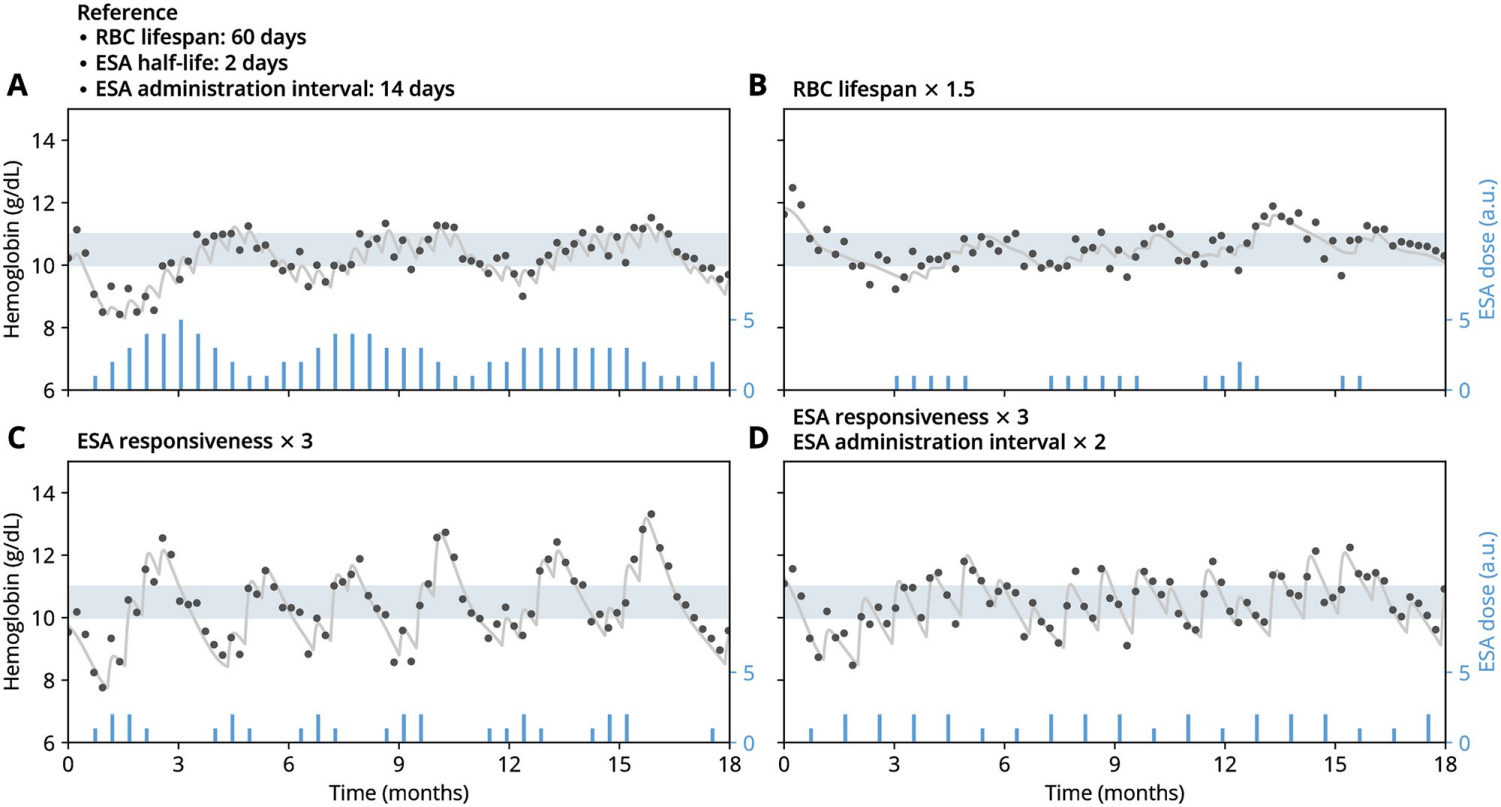
Simulated hemoglobin dynamics display a steady or cycling behavior, respectively, depending on physiological and treatment parameters. Plots show simulated ground-truth hemoglobin levels (gray curves), hemoglobin levels including simulated measurement errors (dots) and ESA administrations (bars). (A) Simulations with a reference parameter set (selected parameters shown above the plot, all other parameters given in Table 1). (B–D) Simulations with parameters varied with respect to the reference parameter set as indicated. In all panels, the shaded region indicates the hemoglobin target window that dose adjustments aim to reach. Initial conditions were drawn from the distribution of equilibrated states. See Methods for simulation details.

To illustrate how this feedback can give rise to Hb cycling, we consider a set of model simulations capturing different treatment modalities and patient physiologies (Fig 3). In a reference scenario (Fig 3A), Hb levels vary mildly around a desired target range, as does the given ESA dose which varies around an average dose needed to keep the patient close to the Hb target range. For a simulated patient with a 50% longer RBC lifespan (Fig 3B), the required ESA dose is substantially lower, while a similar level of Hb variability within the target window is observed. Increasing the patient’s ESA responsiveness (i.e., the magnitude of Hb increase after ESA administration) by a factor of three while keeping all other conditions fixed leads to a pronounced cycling behavior (Fig 3C). Notably, Hb cycles are accompanied by ‘cycles’ in the ESA dose, which mutually maintain themselves in a causal loop. This loop is the quintessential mechanism of self-sustained cycling. In our numerical example, an additional doubling of the ESA administration interval (which is also the dose adjustment interval) leads to the disappearance of the cycling behavior, returning to a mild variation about a steady Hb level (Fig 3D). These examples show that a multitude of treatment- and physiology-related factors contribute to the phenomenon of Hb cycling and the associated ESA administration patterns.

### Hallmarks of self-sustained cycling in clinical data

**Fig 4:**
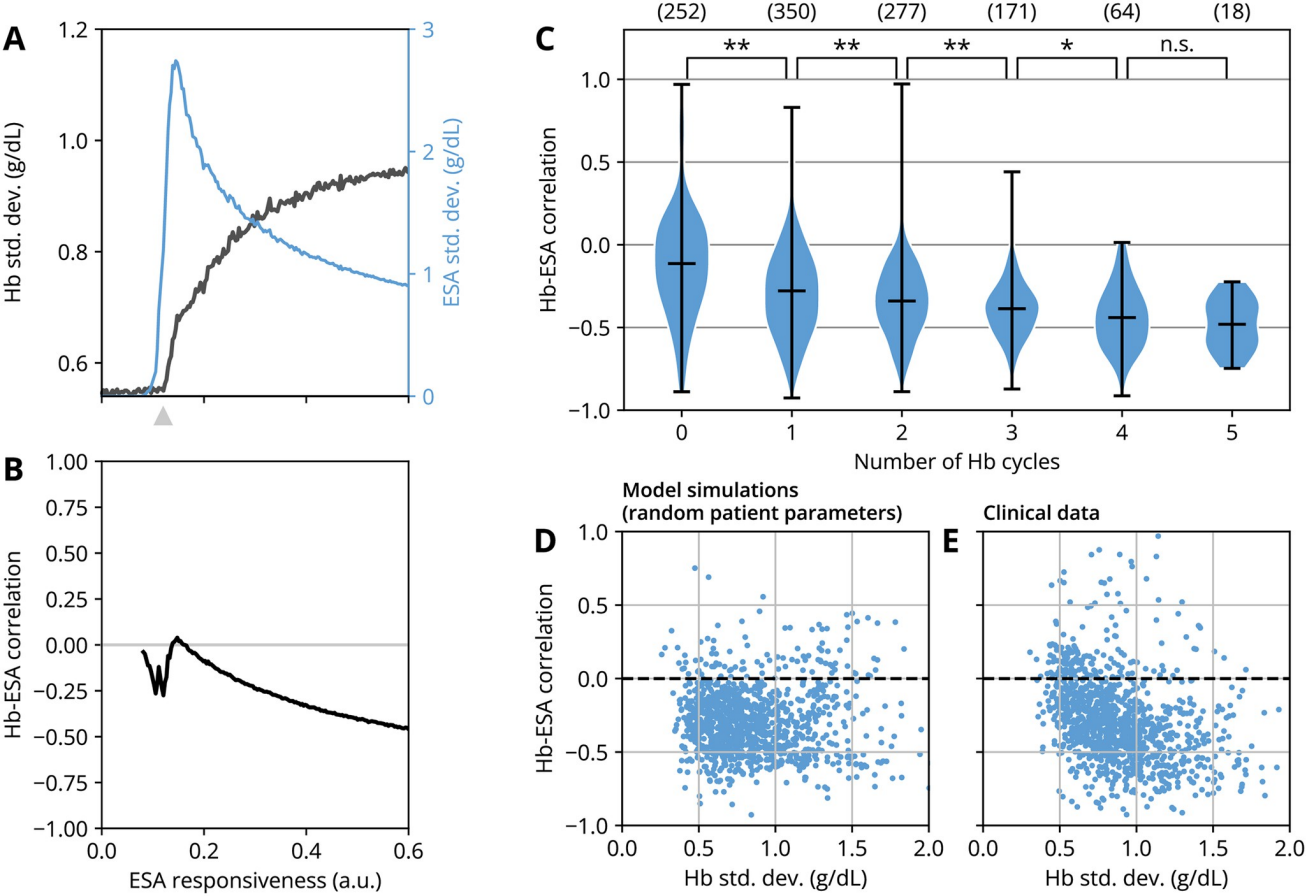
Statistical indicators of self-sustained hemoglobin (Hb) cycling in simulations and in clinical data. (A) The long-term Hb standard deviation (solid curve) serves as a proxy for the amplitude of Hb cycles, shown here as a function of ESA responsiveness in model simulations. The associated ESA standard deviation (blue curve) is displayed for reference. The gray arrow marks the onset of cycling as predicted by the inequality (25). Here, all other model parameters describing patient physiology and treatment modalities, including the dosing interval, are fixed to reference values (1). (B) The Pearson correlation coefficient of Hb levels and ESA doses quantifies the offset (or ‘phase shift’) between bursts of ESA administrations and subsequent Hb cycles and vice versa, see Methods. The correlation coefficient is shown for the same set of simulations as in panel A. (C) Distribution of Hb-ESA correlation coefficients across clinical data, pooled by number of Hb cycles in a patient’s dataset, see Methods. Bars indicate the total range of values, with the center horizontal bar indicating the mean. Numbers in parentheses indicate the number of datasets within the respective group. Pairs of groups were compared using the Anderson–Darling test as indicated (**, *p* < 0.01; *, *p* < 0.05; n.s., *p* > 0.05). (D, E) Pairs of Hb-ESA cross correlation coefficients and Hb standard deviation from (D) model simulations and (E) clinical data (same dataset as in panel C).

To corroborate that this feedback is a typical cause of cycling in clinical treatment, we sought to establish statistical measures for hemoglobin and ESA time series that display characteristic hallmarks of self-sustained cycling. A simple and robust measure for the amplitude of Hb cycles (i.e., that difference between cycle peaks and troughs) is the Hb standard deviation about its mean value. This can be illustrated by, e.g., varying the patient’s ESA responsiveness from the non-cycling to the cycling regime (cf. Fig 3A and 3C) and assessing the Hb standard deviation (Hb sd.) to detect the onset of cycling (Fig 4A). The Hb standard deviation rapidly increases above a critical ESA responsiveness (gray arrow in Fig 4A), indicating the emergence of Hb cycling due to a changed patient physiology if all other treatment modalities are fixed.

To take into account the relation between Hb variability and ESA dosing, we considered the notably coherent offset between the peaks of ESA cycles and Hb cycles in clinical data (Fig 1B) and simulations (Fig 3C). Such an offset (or ‘phase shift’) can be quantified by the Pearson correlation coefficient between ESA dose and Hb levels [27], see Methods. The correlation coefficient ranges from −1 to 1; in the presence of cycling, it indicates whether peaks and troughs of Hb and ESA cycles are perfectly aligned (1) or whether Hb peaks coincide with ESA troughs and vice versa (−1) [28]. A coefficient of 0 may either indicate no correlations (in the absence of cycling) or an offset of half a cycle period (in the presence of cycling). Evaluating the correlation coefficient on the same range of ESA responsiveness values as above, we find that it tends towards more negative correlation coefficients for more pronounced cycling behavior (Fig 4B). This negative correlation implies a characteristic phase shift between ESA cycles and Hb cycles in the presence of self-sustained cycling. Moreover, comparing these two indicators (Hb standard deviation and Hb-ESA correlation coefficient) against each other for this model-based example, we find an association between more pronounced cycling (higher Hb sd.) and a negative correlation between ESA and Hb levels (Fig 4A and 4B).

Next, we sought to detect these statistical signatures in historic clinical time series from in-center hemodialysis patients, see Methods. For each patient time series, we used the total number of algorithmically detected Hb cycles as a proxy for how persistently Hb cycling occurred for a specific patient, see Methods. Pooling Hb/ESA correlation coefficients by the number of detected Hb cycles, we observed that the mean correlation coefficient dropped from slightly below 0 to about −0.5 as the number of detected cycles increased (Fig 4C). Concomitantly, the spread in the distribution of correlation coefficients declined. This indicates the presence of an increasingly coherent phase relationship between ESA dosing and Hb cycling for more persistent Hb cycling, as suggested by the mathematical model.

Independently, we considered the relationship between Hb variability and the Hb-ESA correlation coefficient (Fig 4D and 4E). We used our model to generate simulated long-term patient trajectories for patients with randomly selected physiologies (such as RBC lifespan and ESA responsiveness) and computed Hb sd. and Hb-ESA correlations for each time series (Fig 4D), see Methods for details on the simulation procedure. We did not consider possible correlations between physiological parameters, which are likely present in real patient populations. Despite these simplifying assumptions and the minimal nature of the model, we found a correlation pattern whose trend is remarkably similar to that found in clinical data (Fig 4E): the higher the Hb variability, the more negative the Hb-ESA correlation, with similar quantitative relationships between both quantities in model simulations and clinical data.

We conclude that the studied historic clinical datasets display the characteristic statistical signatures of self-sustained Hb cycling, which were motivated by model predictions. However, it should be noted that within the given datasets, it is impossible to determine the cause of any individual Hb excursion. Apart from the self-sustained cycles studied here, possible causes for cycling are physiological fluctuations and external events such as bleedings and transfusions, as discussed below. We expect that the quantitative deviations of the model results from clinical data and the observed spread in correlation coefficients are due to such random events, the simplistic nature of the model and likely correlations between physiological parameters, which are not considered here.

### Physiological and treatment-related factors promoting hemoglobin cycling

Having established statistical indications for the presence of self-sustained cycling in clinical data, we asked what the main drivers of Hb cycling are. Importantly, the ESA treatment loop (Fig 1A and 1C) comprises (i) long delays between cause and effect (due to, e.g., the long maturation time of RBCs), causing an inert response to perturbations and (ii) a negative feedback (through ESA dose lowerings when Hb levels increase and vice versa). It is well known that negative feedbacks combined with large response delays increase the tendency of a system to cycle and are in many cases design principles of oscillating systems [29].

We first addressed the origin of delays between dose administrations and physiological response (and vice versa) and their role in the emergence of Hb cycles. One of these delays is related to ESA half-life, which can range from about 7 to 140 hours, depending on ESA type and administration route [30]. Long-acting ESAs have to be administered less frequently; on the other hand, accidental misdosings will affect the erythropoietic system for a longer time. The lifespan of red blood cells (RBCs) is another time scale that generates a similar delay: The longer the lifespan the longer excess RBC populations generated by overdosings remain in the system. However, longer RBC lifespans also afford an increased base Hb level, which calls for less extreme ESA dosings (cf. Fig 3A and 3B), thereby stabilizing the system.

**Fig 5:**
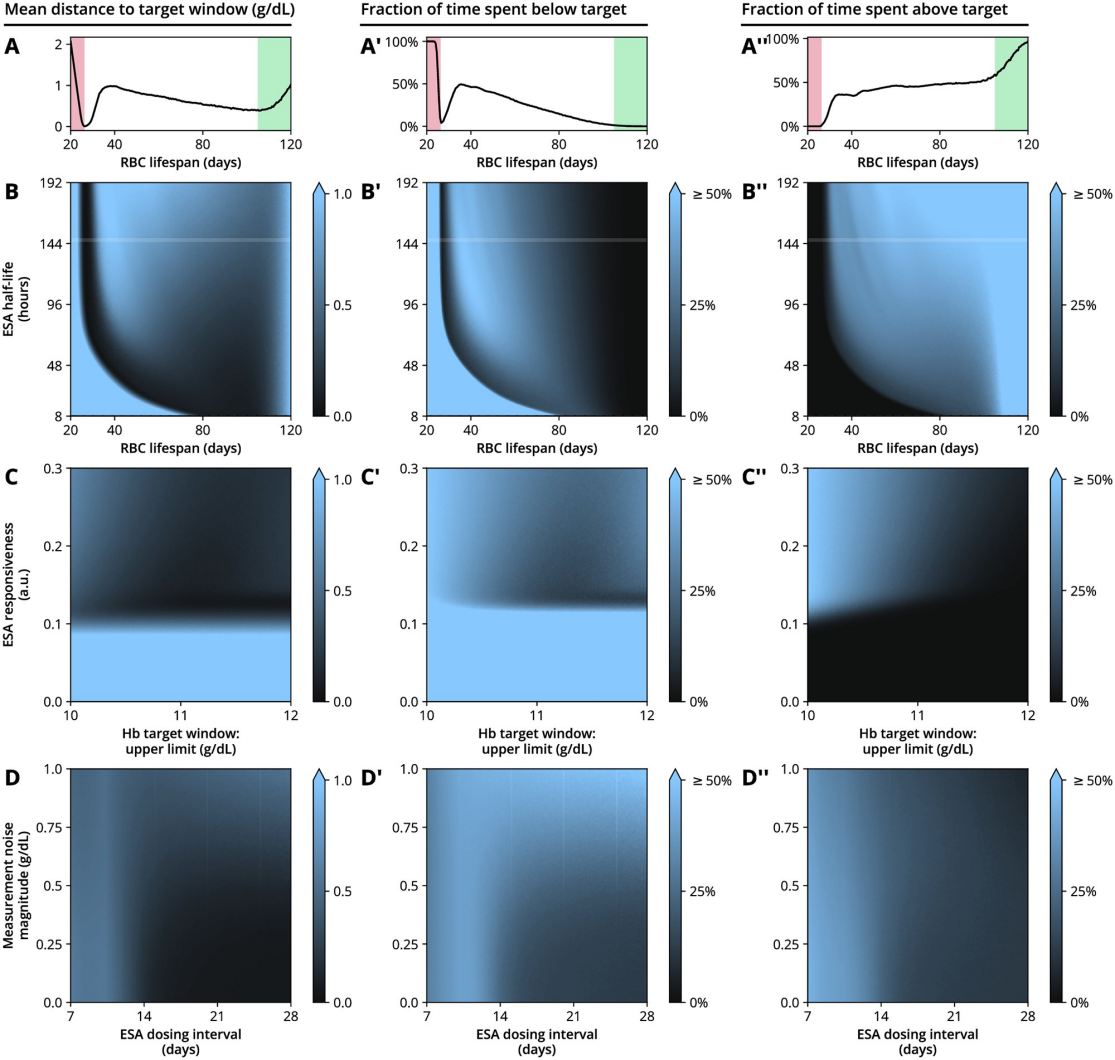
Variability in hemoglobin and ESA administration dynamics for a simulated example patient and treatment setting, where individual physiological and treatment parameters are varied. (A) Mean distance of the ground-truth Hb levels to the specified Hb target range (10–11 g/dL) if RBC lifespan is varied and all other physiological, treatment and ESA parameters are fixed. (A’) Fraction of time the patients spends with Hb levels below the target window and (A”) above the target window as a function of RBC lifespan. In panels A–A”, the red shaded region indicates where RBC lifespan is so small that even repeated administration of the maximum ESA dose cannot achieve Hb target levels, see Eq (15); in the green shaded region, RBC lifespan is high enough to maintain Hb target levels even without ESA therapy, see Eq (16). (B–D”) Contour plots show the indicated quantities if two parameters are varied simultaneously. Columns are organized by quantity displayed and rows are organized by pair of parameters varied. The white line in panels B–B” indicates the cross section of the plot that is displayed in panels A–A”, respectively. Parameters not indicated are fixed to the values provided in Table 1.

**Fig 6:**
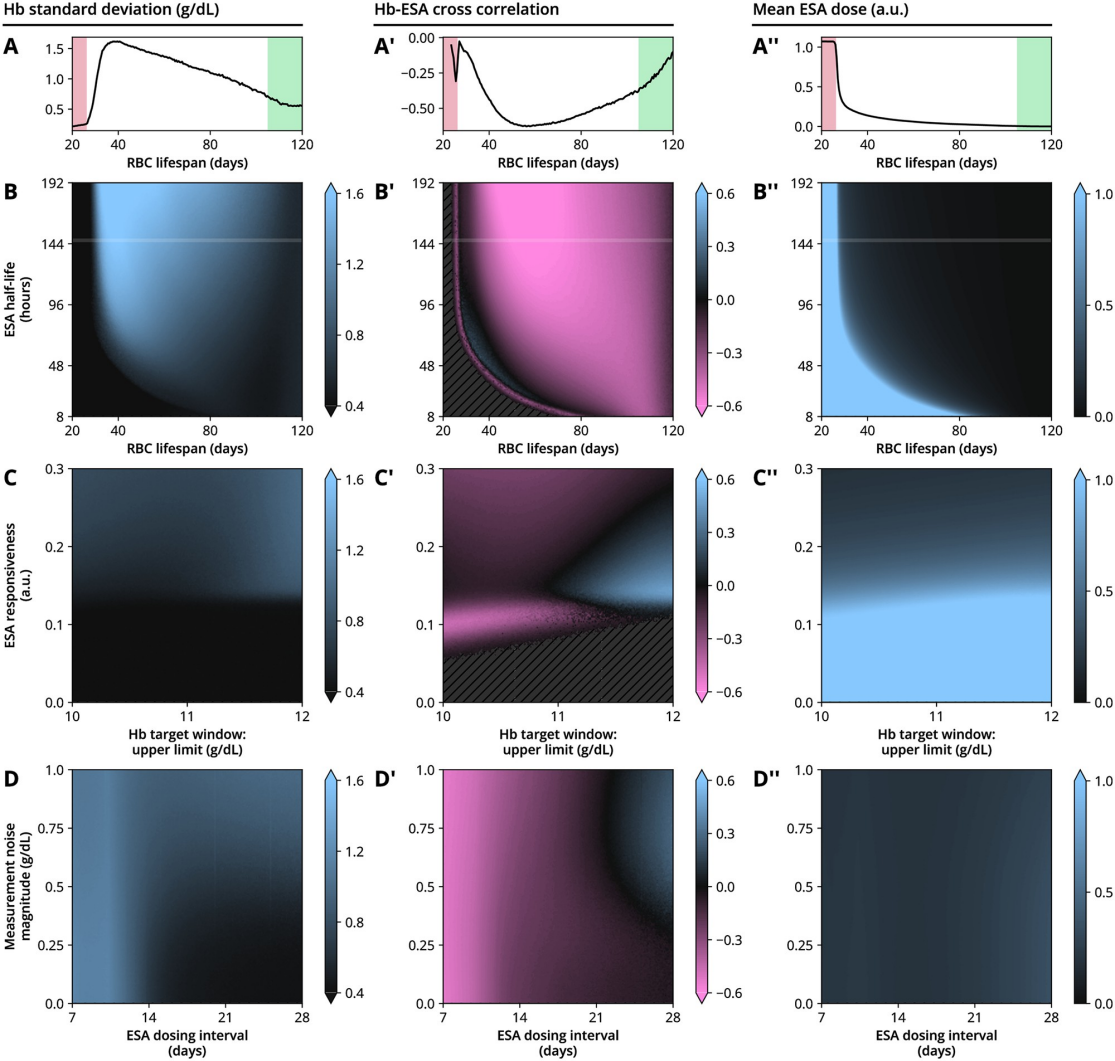
Variability in hemoglobin and ESA administration dynamics for a simulated example patient and treatment setting, where individual physiological and treatment parameters are varied (continued). All conventions identical to Fig 5. (A) Standard deviation of Hb levels, (A’) Correlation coefficient between simulated Hb measurements and ESA doses and (A”) mean ESA dose as a function of RBC lifespan. (B–D”) Contour plots show the indicated quantities if two parameters are varied simultaneously. Columns are organized by quantity displayed and rows are organized by pair of parameters varied. Hatched regions in panels B’ and C’ indicate parameter regions where the Hb-ESA correlation coefficient is not defined. Parameters not indicated are fixed to the values provided in Table 1.

To study the interplay between these time scales, we systematically conducted model simulations varying selected parameters, while keeping all other parameters fixed to reference values (Figs 5 and 6). We were particularly interested in (i) the ability of the system to stabilize Hb levels within the chosen target range of 10–11 g/dL, as indicated by the mean distance of simulated Hb measurements to the target window (from below or above) and the average time spent below and above the target window (Fig 5), (ii) whether large Hb variability (including cycling) occurred, as measured by the Hb standard deviation and the Hb-ESA correlation introduced above (Fig 6A–6D and 6A’–6D’) and (iii) how this is associated with average ESA usage (Fig 6A”–6D”).

Model simulations revealed that very low RBC lifespans lead to Hb levels not reaching the target range (even if the maximum allowed ESA dose is repeatedly administered) (red shaded regions in Fig 5A–5A”), resulting in higher ESA usage (Fig 6A”). In this region of low RBC lifespans, self-sustained Hb cycling is absent as indicated by small Hb standard deviations (Fig 6A and 6B). On the other hand, long RBC lifespans (i.e., close to the 120 days observed in healthy humans) lead to increased Hb base levels and make it more likely that the mean Hb concentration lies within or above the chosen target range (green shaded regions Fig 5A–5A”). This case may require only mild or no ESA therapy, decreasing the tendency of the system to display Hb cycling with a considerable amplitude (Fig 6A and 6B). The two limiting cases of very low and healthy RBC lifespans can be analytically located using a model approximation (see Eqs (15) and (16) and their derivation in the Methods section). Between long and very short RBC lifespans, the model exhibited a region in which Hb cycling is facilitated, associated with lowered Hb levels (around an RBC lifespan of 40 days and sufficiently large ESA half-life in the numerical examples in Figs 5A–5B” and 6A–6B”). In this region, depending on other time scales such as ESA half-life, the system is too inert to immediately recover from perturbations but also not inert enough to retain enough RBCs by itself. This behavior can be studied systematically by an analytical investigation of the oscillatory behavior of the model, see Methods. The existence of such ‘peninsulas’ of Hb cycling in parameter space (like the one visible as the bright region in the top of Fig 6B) suggests that the knowledge of individual physiological properties of anemia patients may be of limited use when it comes to estimating the tendency to display Hb cycling. Between these two regimes, there is a ‘sweet spot’ (dark regions in Fig 5B–5B”), where the ESA remains long enough in the system to benefically promote erythropoiesis without enabling self-sustained cycling. We emphasize that the location of this ‘sweet spot’ depends on other parameters describing patient physiology, treatment scheme and Hb target window; Figs 5 and 6 show numerical examples where all but two parameters were fixed.

We also simulated the effects of varying ESA responsiveness (i.e., the magnitude of the Hb response evoked by a given ESA dose) and the width of the Hb target window, ranging from 10 g/dL on the lower end to a variable upper end, with 11 g/dL being the reference scenario. While too low ESA responsiveness sensibly leads to Hb levels consistently below the target window (Fig 5C’), increasing responsiveness beyond a viable point tends to facilitate cycling (Fig 6C), consistent with previous clinical observations [5]. Sensibly, a wider target window generally leads to more time being spent within the target window (Fig 5C–5C”). However, this is only the case up until a point where the upper limit of the target window is sufficiently far away from the ESA hold limit, which is located at 12 g/dL in our reference scenario. When the target range approaches the hold limit, frequent small Hb fluctuations or measurement errors will yield Hb measurements above the hold limit and therefore lead to an abrupt stop of ESA administrations (Fig 2). Since ESA doses may only be increased by discrete steps, it may take several administration cycles until the ESA dose has reached again the level necessary to maintain a patient’s Hb levels within (or around) the target window. During this readjustment phase, Hb levels rise together with the ESA dose, a fact reflected by a positive correlation between Hb levels and ESA doses (blue region in Fig 6C’), as opposed to the negative correlation seen during Hb cycling (e.g., magenta region in Fig 6B’). (Interestingly, in this parameter regime, the time course of ESA doses bears similarity with so-called ‘Sisyphus random walks’ from renewal theory, whereby constant growth of a process is interrupted by stochastic resetting [31]).

Finally, we investigated the role of Hb measurement errors (or ‘measurement noise’) in combination with different ESA dosing intervals. Increased measurement noise lead to increased variability in ground truth Hb levels, which unlike Hb measurements, are only indirectly affected by measurement noise as erroneous measurements have a higher probability of entailing misdosing events based on these measurements (Fig 6D). The ESA dosing interval plays a double role since it constitutes the delay between successive dose administrations on one hand and dose adjustments on the other hand and therefore mitigates the effects of large dose changes. In simulations, longer ESA dosing intervals are found to be beneficial for stabilizing the Hb dynamics within the target window (Fig 6D). Such a behavior is supported by analytical arguments, see the derivation of the inequality (25) in the Methods section. This observation is compatible with reports of an increased month-to-month hemoglobin variability and ESA dose variability under a biweekly ESA administration scheme as compared to a four-weekly scheme, observed in a randomized controlled trial with hemodialysis patients for both CERAs and Darbepoetin alfa [32]. On the other hand, the ESA administration interval is clearly bounded from above as the ESA half-life sets the time scale on which a next administration is necessary to maintain Hb levels within the target range. Together, these arguments suggest the existence of a patient- and ESA-dependent ‘sweet spot’ for the ESA administration interval as well.

The above considerations show how stabilization of Hb levels and the occurrence of sustained Hb cycling depend on a multitude of physiological and treatment-related factors and provide an explicit explanation why it is difficult to identify a single treatment design that accommodates different patient physiologies.

### Transient cycling induced by perturbations

Next to such systemic drivers of cycling, internal and external perturbations of the system may trigger additional transient cycles. In a clinical context, such perturbations can be singular events such as bleedings, blood transfusions, missed or erroneous doses or a change of medication type on one hand, or recurrent fluctuations introduced by short-term lifestyle changes and suboptimal dosing decisions due to Hb measurement errors on the other hand. In general, the larger the tendency for self-sustained (i.e., persistent) Hb cycling to occur, the more likely the emergence of transient cycles through such external events and the longer the time it takes for such transient cycling to decay (see Methods).

**Fig 7:**
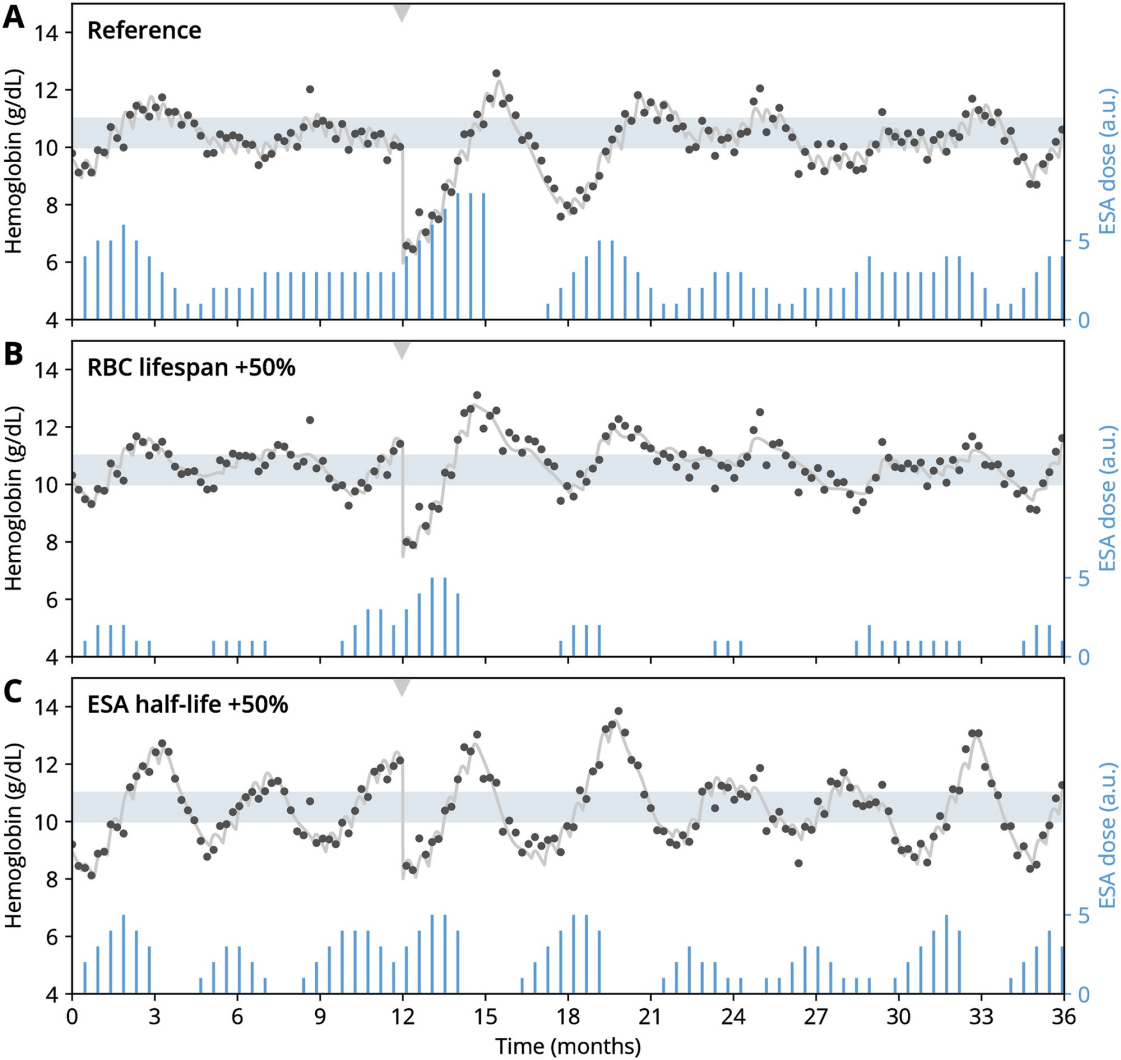
Transient cycling can be induced by events unrelated to treatment such as sudden blood loss. (A–C) Simulated bleeding scenarios where hemoglobin levels have been instantly reduced by 4 g/dL at 12 months (gray arrow). Parameter values for the reference scenario (panel A) are provided in Table 1. Parameter changes in panels B and C as indicated in the plot. All display conventions as in Fig 3.

Such a differential behavior can be illustrated by simulating a blood loss scenario with our model. Blood loss elicits, among others, the refilling of the blood volume with interstitial fluid redistributed from the extravascular space on the time scale of minutes [33, 34]. This leads to a quick decline of the blood hemoglobin concentration, which can be mimicked in simulations (Fig 7). Depending on the hematological state of the patient at the time of the bleeding, their physiological parameters (such as RBC lifespan) as well as treatment parameters (such as ESA half-life), such a blood loss event may either lead to pronounced transient cycling that decays over an extended time (Fig 7A), a quick return to the target window without prolonged cycling periods (Fig 7B) or the maintenance or induction of sustained cycling in systems where both steady states and sustained cycling behaviors may be found (Fig 7C). This illustrates that while Hb cycling can be triggered and initially driven by external (and from a treatment perspective ‘random’) events, it is the feedback loop between a patient’s physiological response to ESA treatment and the treatment design that determines for how long such perturbations affect the system after their occurrence.

## Discussion

Hemoglobin cycling can be frustrating to the practicing nephrologist. Despite best efforts, knowledge of and adherence to dosing schedules, guidelines, and state-of-the-art literature, hemoglobin cycling can emerge unexpectedly, without discernible cause. The clinical practitioner may attribute hemoglobin cycling to ESA dosing schedules, intercurrent events (e.g., occult hemorrhage, infections, and hospitalizations), missed ESA dosing, or incorrect hemoglobin labs (especially in patients with central venous catheter as vascular access). When confronted on the bed side with hemoglobin cycling, trial-and-error interventions, such as changing the ESA type (e.g., to an ESA with a different half-life) and modifying ESA dosing intervals, may ensue. Unfortunately, the root cause of hemoglobin cycling may remain elusive as important factors, such as RBC lifespan, are unknown.

Our research was motivated by the pursuit of clarity through the development of a minimal model that could explain essential characteristics of hemoglobin cycling. We are aware that a minimal model cannot—by definition—explain the complex impact of clinical and stochastic events that may trigger or exacerbate hemoglobin cycling. Our goal was increased clarity through abstraction by applying a careful, reductionist approach. The resulting minimal model could then serve as a building block for future, more complex models. Using the model, we have shown how self-sustained hemoglobin (Hb) cycling occurs as a consequence of the feedback between a patient’s physiological response to ESA administrations and the resulting dose adjustments. Despite its simplicity, the model is able to predict the trends of statistical key features of hemoglobin cycling in clinical data such as the temporal correlations between ESA dose administrations and Hb levels and their association with Hb variability and cycling. This corroborates that the proposed mechanism of cycling accounts for a considerable portion of clinically observed Hb cycling periods.

Investigating the model behavior in various parameter regimes shed light on the multitude of factors determining the success and the shortcomings of ESA treatment: First, with regard to Hb dynamics, ESA-specific properties such as its half-life may have a ‘sweet spot’ within which ESA is retained sufficiently long in the body to elicit a substantial effect and short enough to not facilitate Hb cycling. Second, some patient- and therapy-related factors may affect the Hb dynamics in multiple (sometimes competing) ways, making their overall effect unobvious. This includes a long RBC lifespan (which both affords higher average Hb levels and leads to a prolonged effect of misdosing events) and the ESA dosing interval (which typically sets the time scale of both dose administrations and dose adjustments). Our model suggests that longer RBC lifespans generally have a favorable effect in requiring less ESA on average and preventing cycling. Longer ESA dosing intervals lead to a lowered average ESA usage and tend to inhibit cycling in the model; on the other hand, the maximum possible dosing interval is clearly constrained by ESA half-life, determining the speed with which the effects of an ESA administration decay. Given the physiological heterogeneity of anemia patients, the above considerations manifest why adjusting a general ESA treatment algorithm for an entire patient population is notoriously difficult. For instance, RBC lifespan is known to vary strongly among CKD patients [35], in contrast to healthy subjects; this may be one explanation why, under the same ESA treatment regimen, some CKD patients exhibit Hb cycling while others do not.

The above general insights illuminate the emergence of Hb cycling and may aid in the development of improved population-level or personalized ESA treatment algorithms. The minimal model presented here is a framework for testing general trends when altering treatment modalities. For example, it is likely that treatment algorithms considering not only most recent Hb measurements but longer-term trends will be able to provide much better dosing decisions; such schemes can be captured by straightforward extensions of the present model. The performance of the resulting algorithms can then be systematically tested via computational frameworks that simulate anemia management under real-world conditions using ‘virtual patient populations’ [23]. Novel developments in the pharmacologic treatment of anemia such as hypoxia-inducible factor prolyl hydroxylase inhibitors (HIF-PHIs), which stimulate endogenous EPO expression via the HIF signaling pathway, may also profit from these insights as the model can be easily adapted to the much different dosing frequencies of HIF-PHIs (typically several times a week).

Beyond anemia, the current model presents a template for investigating the features of chronic disease therapies that aim to replace an impaired or failing homeostatic feedback within the body. This applies in particular to cases where there are considerable delays between drug administration and physiological response and where patients are treated to target as monitored by a clinical key parameter (such as blood hemoglobin concentration in the case of anemia). This is the case in warfarin treatment to regulate blood coagulation, monitored by prothrombin time including corresponding target values, iron therapy (both via the oral and the intravenous route) in the case of functional iron deficiency, supplemental nutrition in the case of albumin deficiency and 1*α*,25-Dihydroxyvitamin D3 (1,25D3) and calcimimetic drug therapy to treat secondary hyperparathyroidism.

## Methods

### Ethics statement

Patient data were collected in the course of a clinical research project that has received Institutional Review Board approval (Protocol #039–08, Institutional Review Board Beth Israel Medical Center, New York, NY, USA). Written patient consent was obtained for the clinical research protocol #039–08. Secondary analysis of data obtained under protocol #039–08 was determined as exempt from review by the Western Institutional Review Board, Puyallup, WA, USA (protocol #ES-20–003).

### Patient datasets

In total, 3298 historic time series from in-center hemodialysis patients were considered. The time series comprised weekly Hb measurements as well as comprehensive recordings of ESA administrations. Of those, 1134 time series satisfied the following criteria for the computation of a correlation coefficient (see ‘Hemoglobin-ESA correlation coefficient’): (i) at least 5 ESA administrations in the recorded time interval, (ii) no gaps in Hb recording for more than 30 days, (iii) finite normalized cross correlation (requires non-vanishing variance in ESA doses). The datasets had a median length of 448 days with an interquartile range of 399–448 days.

### Hemoglobin-ESA correlation coefficient

Time points of Hb measurements and ESA dose recordings may not overlap, so that we used a spline interpolation of Hb datapoints to compute Hb levels at the points of ESA administration. Spline interpolation was performed using the SciPy function splrep [36] with smoothness parameter given by s = 0.2 × (number of datapoints). Moreover, since only non-zero ESA doses were explicitly recorded, an imputed ESA time series was generated through the following procedure: Whenever the time interval between two successive ESA administrations was larger than 14 days (the median time between successive ESA dose administrations), it was filled with zeroes at a distance of 14 days starting from the last non-zero dose. This method ensures that ‘zero doses’ enter the cross correlation as well. Denoting by *h* = (*h*_1_, …, *h*_*n*_) the interpolated Hb time series evaluated at the times of the imputed ESA dose history *x* = (*x*_1_, …, *x*_*n*_), the Pearson correlation coefficient is given by

$$\begin{array}{c} C\left[h,x\right]=\frac{\left\langle hx\rangle -\langle h\rangle \langle x\right\rangle }{\sqrt{\left\langle h^{2}\right\rangle -{\left\langle h\right\rangle }^{2}}\sqrt{\left\langle x^{2}\right\rangle -{\left\langle x\right\rangle }^{2}}}\;, \end{array}$$

where $\langle x\rangle =n^{-1}\sum_{i=1}^{n}x_{i}$ denotes the average of a time series *x*. The analogous definition for a continuous time series *x* = *x*(*t*) is given by replacing the average by $\langle x\rangle =T^{-1}\int_{0}^{T}x\left(t\right)\;dt$, where *T* is the averaging time. For sinusoidal, phase-shifted signals *a*(*t*) = sin(*ωt*), *b*(*t*) = sin(*ωt* + *δ*), the correlation coefficient is given by *C*[*a*, *b*] = cos *δ*, so that in the presence of oscillations, *C* carries information about the phase shift between time series.

### Number of hemoglobin peaks

The number of Hb peaks within a time series was determined using the same interpolated Hb time series used to calculate the Hb/ESA correlation coefficient. Peaks were detected through the SciPy function find_peaks with parameter peak_prominence = 0.75 [36].

### Minimal model of hemoglobin cycling

We introduce a dynamical model that describes the closed feedback between patient physiology and erythropoiesis-stimulating agent (ESA) treatment response. While this model is highly simplified it captures the essential elements contributing to therapy-induced cycling. The model comprises three dynamic variables representing the patient’s hemoglobin (Hb) levels (*h*), the ESA dose prescription (represented by a discrete index *x* indicating an ESA dose in a dosing chart) and the patient’s systemic ESA concentration (*e*). In the absence of ESA treatment, hemoglobin levels tend towards a (lower than healthy) steady-state level that results from the balance of RBC production (at a net rate λ) and eryptosis (at a rate *η*). ESAs upregulate the patient’s Hb levels with a given patient-specific responsiveness *μ* and a time delay *τ* after administration, which reflects the typical relevant proliferation and maturation times of the erythropoietic lineage. Systemic ESA levels decay at a rate *κ* related to the ESA half-life. The prescribed ESA dose is updated every time interval *τ*_int_ based on the patient’s Hb levels, followed by an ESA administration directly afterwards. The ESA dose is capped at a maximum value *x*_cap_ representing the highest available single dose. The corresponding system of dynamic equations is given by

$$\begin{array}{c} \dot{h}=\lambda +\mu \phi (e_{\tau })-\eta h\;, \end{array}$$ (1)

$$\begin{array}{c} \dot{x}=\Delta \left(t+0^{+}\right)\times \{\begin{array}{cc} f(x,h) & x\leq x_{\text{cap}}\;\text{or}\;f\left(x,h\right)\leq 0\\ 0 & \text{else} \end{array}\;, \end{array}$$ (2)

$$\begin{array}{c} \dot{e}=c(x)\Delta (t)-\kappa e\;. \end{array}$$ (3)

where *e*_*τ*_ (*t*) = *e*(*t* − *τ*) denotes the delayed variable. Due to the presence of this delay, Eqs (1)–(9) constitute a system of delay differential equations, for which an initial history of length *τ* (rather than an initial condition at a single point in time) has to be specified to uniquely determine its solution.

Furthermore, *ϕ* denotes a sigmoidal function of the Hill type, which describes a nonlinear dose-response relationship and accounts for the fact that there is little effect of systemic ESAs below a minimal effective concentration,

$$\begin{array}{c} \phi \left(e\right)=\frac{e^{n}}{{\bar{e}}^{n}+e^{n}}\;. \end{array}$$ (4)

Here, *n* denotes the Hill exponent which determines the steepness of the sigmoidal feedback and $\bar{e}$ denotes the half maximal effective concentration (EC_50_) of the ESA. In Eq (1), we identify ESA half-life and RBC lifespan as

$$\begin{array}{c} T_{\text{ESA}}=\frac{\text{ln}\;2}{\kappa }\;,\;T_{\text{RBC}}=\frac{1}{\eta }\;. \end{array}$$ (5)

The function *f*(*x*, *h*) determines how the prescribed ESA dose is altered according to the current Hb level *h* and the currently prescribed dose *x*. The function *c* defines the dosing chart. For simplicity, we use a dosing chart in which the given dose is a multiple of a given minimal dose *c*_0_,

$$\begin{array}{c} c\left(x\right)=c_{0}x\;, \end{array}$$ (6)

where *x* is a positive integer. Periodic adjustment of the dose and administration is described by the function

$$\begin{array}{c} \Delta \left(t\right)=\sum_{n}\delta \left(t-n\tau_{\text{int}}\right)\;, \end{array}$$ (7)

where *δ* is the Dirac delta distribution. The term 0^+^ in Eq (2) indicates that the function Δ is evaluated slightly before Δ in Eq (3) to ensure that the prescribed dose is updated before the administration. Here, we consider a simple dose adjustment scheme in which the dose is increased/decreased if Hb levels are below/above a target window, specified by the lower and upper bounds *h*_−_ and *h*_+_, respectively. Furthermore, the dose is set to zero if Hb levels rise above a critical ‘hold limit’ *h*_hold_. A decision tree corresponding to this dosing scheme is shown in Fig 2. This decision tree can be expressed through the function *f* as follows,

$$\begin{array}{c} f(x,h)=\{\begin{array}{cc} g(x,h) & x>0\\ \text{max}\;(g(x,h),0) & x=0 \end{array}\;, \end{array}$$ (8)

$$\begin{array}{c} g(x,h)=\{\begin{array}{cc} \Theta \left(h_{+}-h\right)+\Theta \left(h_{-}-h\right)-1 & h<h_{\text{hold}}\\ -x & h\geq h_{\text{hold}} \end{array}\;, \end{array}$$ (9)

where Θ is the Heaviside step function. The case distinction in the definition of *f* ensures that the dose index cannot become negative; the distinction in the definition of *g* imposes a ‘hold’ limit, i.e., a dose decrease to zero if the Hb levels exceed at limiting amount *h*_hold_.

Especially in patients with renal disease, Hb levels are affected by random perturbations such as changes in fluid status depending on patient activities that are uncorrelated with ESA treatment. Moreover, Hb measurements usually suffer from measurement errors introduced by a variety of preanalytical and analytical variabilities. In the model, the effect of uncorrelated Hb perturbations and Hb measurement errors can be incorporated through additional random variables, modulating Hb dynamics and ESA dosing decisions. Here, we include these stochastic components through the replacements

$$\begin{array}{c} \dot{h}\to \dot{h}+\omega_{1}\xi_{1}\left(t\right)\;, \end{array}$$ (10)

$$\begin{array}{c} f(x,h)\to f(x,h+\omega_{2}\xi_{2}\left(t\right))\;, \end{array}$$ (11)

in Eqs (1) and (2), where *ω*_1_ and *ω*_2_ denote the noise strength of physiological and measurement noise, respectively, and where *ξ*_1_ and *ξ*_2_ are random functions with zero mean, fluctuating at a characteristic time scale. The random functions *ξ*_*i*_ were realized as polynomial interpolations of normally distributed random numbers with zero mean and unit variance, dispersed on an equidistant grid with time spacing Δ*t*_*i*_. The distance between grid points determines the characteristic correlation time of the respective random function.

### Simulation procedure for Fig 4D

Each point in the scatter plot in Fig 4D was computed from one realization of the model, with the patient-dependent parameters λ, *η*, *μ*, *κ* and *ω*_1_ set to the random value *ζv*_*i*_, where *ζ* is a random number drawn from the positive part of the normal distribution N(1,1/3) and *v*_*i*_ is the reference parameter value of parameter *i*, see Table 1. Lengths of the simulated time series were randomly chosen from the distribution of the lengths of clinical time series from which Fig 4E was generated. Simulations started in a state drawn from the equilibrium distribution for the respective parameter set.

**Table 1:** List of reference model parameters.

Parameter	Unit	Ref. value	Description	Model part
λ	g dL^−1^ d^−1^	0.1	Hb base gain rate	Eq (1)
*μ*	g dL^−1^ d^−1^	0.3	ESA responsiveness	Eq (1)
$\bar{e}$	a.u.	0.75	ESA EC_50_	Eq (4)
*n*	1	2	Hill exponent for the Hb response	Eq (4)
*τ*	d	10	Delay between ESA adminstration and Hb response	Eq (1)
*η*	d^−1^	1/60	Eryptosis rate ($\eta =T_{\text{RBC}}^{-1}$)	Eq (1)
*x*_cap_	a.u.	15	Maximum available ESA dose	Eq (2)
*κ*	d^−1^	0.36	ESA clearance rate ($\kappa =\left(\text{ln}\;2\right)T_{\text{ESA}}^{-1}$)	Eq (3)
*τ*_int_	d	14	Time interval between consecutive dose administrations	Eq (7)
(*h*_−_, *h*_+_)	g dL^−1^	(10, 11)	Hb target window	Eq (9)
*h*_hold_	g dL^−1^	12	‘Hold limit’; Hb threshold above which ESA is held off	Eq (9)
*c*_0_	a.u.	1	ESA dose step, minimal non-zero dose	Eq (9)
*ω*_1_	g dL^−1^ d^−1^	0.02	Physiological noise strength	Eq (10)
Δ*t*_1_	d	14	Physiological noise interpolation grid distance	Eq (10)
*ω*_2_	g dL^−1^	0.4	Measurement noise strength	Eq (11)
Δ*t*_2_	d	5	Measurement noise interpolation grid distance	Eq (11)

### Onset of cycling and characterization of cycling solutions

#### Model approximation with smooth dynamics

To obtain analytical insights into the phase behavior of the system, we resort to an approximated (‘smoothed’) version of the model Eqs (1)–(3) in which all discrete jumps in the dynamics are replaced by smooth functions, the hold limit is removed and the constraints on the dose index are released. While such a simplified model cannot be expected to capture any potential effects of the discrete nature of ESA dosing on the onset, period and amplitude of oscillations, it is a useful tool to study the qualitative contributions of different effects within the physiologically plausible parameter regime. Importantly, as shown below, it demonstrates that the discreteness of ESA administrations is not a necessary factor to induce Hb cycling (while it may contribute to it).

In the smoothed model, the following features are approximated:

- We consider a continuous dose administration scheme at an average rate $\Delta \left(t\right)=\tau_{\text{int}}^{-1}$, and a continuous dose adjustment algorithm, $f\left(x,h\right)=-\text{tanh}\;(\left[h-\bar{h}\right]/\sigma )$, where $\bar{h}=\left(h_{+}+h_{-}\right)/2$ denotes the center of the Hb target window and *σ* = (*h*_+_ − *h*_−_)/2 denotes half the width of the target window. Here, the tanh function serves as a smoothed step function with properties similar to the function *g* in Eq (9).
- As a consequence of these replacements, there are no cap on the maximum ESA dose and no hold limit in the smoothed model.
- The dose variable *x* can take any real value.

Hence, if probed in implausible parameter regimes, these simplifications can lead to unphysiological behavior such as negative doses and concentrations. For the dosing chart *c*, we use the simple linear scheme given by Eq (6). Altogether, the approximated dynamics is given by

$$\begin{array}{c} \dot{h}=\lambda +\mu \phi \left(e_{\tau }\right)-\eta h\;,\;\;\dot{x}=-\frac{1}{\tau_{\text{int}}}\text{tanh}\;(\frac{h-\bar{h}}{\sigma })\;,\;\;\dot{e}=\frac{c_{0}}{\tau_{\text{int}}}x-\kappa e\;. \end{array}$$ (12)

#### Steady state

The system Eqs (12) has a steady state (for which $\dot{h}=0$, $\dot{x}=0$, $\dot{e}=0$) that corresponds to a repeatedly administered ESA dose x_*_ which keeps the patient at the target level $\bar{h}$ and steady systemic ESA levels e_*_,

$$\begin{array}{c} \begin{array}{c} h_{*}=\bar{h}\;,\;x_{*}=\frac{\kappa \tau_{\text{int}}}{c_{0}}e_{*}\;,\;e_{*}=\phi^{-1}\left(E\right)\;, \end{array} \end{array}$$ (13)

where $E=(\eta \bar{h}-\lambda )/\mu$. Here, *ϕ*^−1^ denotes the inverse of *ϕ*, which exists because *ϕ* is monotonic for positive arguments. Expressing the steady-state ESA dose through the definitions given by Eq (5), we obtain

$$\begin{array}{c} E=\frac{\bar{h}/T_{\text{RBC}}-\lambda }{\mu }\;. \end{array}$$ (14)

Since *ϕ*^−1^ is monotonically increasing, this shows that ESA consumption increases with decreasing RBC lifespan (*T*_RBC_) and decreasing ESA responsiveness (*μ*).

Note that *ϕ*^−1^(*E*) is defined only on the value range of *ϕ*, i.e., for 0 < *E* < 1, which has a physiological interpretation that can be expressed, e.g., as conditions on RBC lifespan via Eq (14). The criterion *E* > 0 translates into

$$\begin{array}{c} T_{\text{RBC}}<\frac{\bar{h}}{\lambda }\;. \end{array}$$ (15)

Violation of this criterion corresponds to cases where the body’s residual capacity to generate and maintain RBCs leads to hemoglobin levels above the target level $\bar{h}$ even in the absence of ESA therapy. Thus, within the model paradigm, the inequality (15) ensures that ESA therapy is necessary. Likewise, the criterion *E* < 1 translates into

$$\begin{array}{c} T_{\text{RBC}}>\frac{\bar{h}}{\mu +\lambda }\;. \end{array}$$ (16)

Violation of this criterion corresponds to the case where eryptosis proceeds so quickly that hemoglobin target levels cannot be reached even if the dose is arbitrarily increased, due to the saturating effect of the ESA.

#### Stability of the steady state

Whether the system is able to remain at its steady state depends on whether small perturbations to the steady state decay over time or are amplified. Stability of the equilibrium can be assessed through a standard linear stability analysis [37]: By **v** = (*h*, *x*, *e*) we denote the vector of model variables and by v_*_ = (h_*_, x_*_, e_*_) the steady state given by Eq (13). Linear stability is probed through the ansatz **v**(*t*) = v_*_ + *ε***u**e^*αt*^, where *ε***u** is a small perturbation with *ε* being an expansion parameter and *α* is the growth exponent of the perturbation; the steady state is stable if the real parts of all solutions for *α* are negative. Using this ansatz in Eqs (12), yields to first order in *ε* the equation *α***u** = **J**(*α*)**u**, where

$$\begin{array}{c} J\left(\alpha \right)=(\begin{array}{ccc} -\eta & 0 & \mu^{\prime }e^{-\alpha \tau }\\ -{\left(\sigma \tau_{\text{int}}\right)}^{-1} & 0 & 0\\ 0 & \tau_{\text{int}}^{-1}c_{0} & -\kappa \end{array})\;, \end{array}$$ (17)

and

$$\begin{array}{c} \mu^{\prime }=\mu \phi^{\prime }\left(e_{*}\right)\;. \end{array}$$ (18)

Hence, solutions for the exponent *α* are eigenvalues of **J**, corresponding to the (generally complex) roots of the characteristic function *χ*(*α*) = det(*α***I** − **J**(*α*)), where **I** is the identity matrix. The characteristic function is given by

$$\begin{array}{c} \chi \left(\alpha \right)=\beta e^{-\alpha \tau }+\alpha \left(\alpha +\kappa \right)\left(\alpha +\eta \right)\;, \end{array}$$ (19)

where

$$\begin{array}{c} \beta =\frac{\mu^{\prime }c_{0}}{\sigma \tau_{\text{int}}^{2}}\;. \end{array}$$ (20)

Since *χ*(*α*) = 0 is a transcendental equation due to the presence of the delay *τ*, no closed form for its solutions is available. However, candidates for self-consistent approximations can be found by truncating *χ* at second order in *α*,

$$\begin{array}{c} \chi \left(\alpha \right)=\beta +\gamma \alpha +m\alpha^{2}+O\left(\alpha^{3}\right)\;, \end{array}$$ (21)

where we have defined

$$\begin{array}{c} \gamma =\kappa \eta -\beta \tau \;,\;m=\kappa +\eta +\frac{\beta \tau^{2}}{2}\;. \end{array}$$ (22)

The corresponding roots are given by

$$\begin{array}{c} \alpha_{\pm }\approx -\frac{\gamma \pm \sqrt{\gamma^{2}-4\beta m}}{2m}\;. \end{array}$$ (23)

This amounts to approximating the linearized dynamics in the vicinity of the steady state by a damped harmonic oscillator of the type $m\ddot{z}+\gamma \dot{z}+\beta z=0$, where *z* is the oscillating variable (the perturbation about the steady state), *m* corresponds to the inertial parameter (analogous to a ‘mass’), *γ* to the damping coefficient and *β* to the restoring force strength (analogous to the ‘spring constant’ of a Hookean spring). Generally, the smaller the damping and the larger the restoring force, the more susceptible the system is for oscillations.

Interestingly, the ‘spring constant’ *β*, given by Eq (19), comprises all treatment-related parameters (the base ESA dose *c*_0_ of which all higher doses are a multiple, Hb target window width *σ* and dosing interval *τ*_int_) as well as the ESA responsiveness *μ* and therefore represents all treatment-related contributions to the stability of the steady state. This reflects the fact that the treatment algorithm acts as ‘restoring force’ to deviations from the hemoglobin set point. Notably, the administration interval *τ*_int_ enters quadratically, reflecting the fact that this same time scale appears as both the dose adjustment interval and the administration interval. Moreover, most physiological and treatment-related parameters appear in both composite parameters *m* and *γ*. In our harmonic oscillator analogy, this implies that the same physiological or treatment-related feature can affect both the inertia of the system (which favors cycling behavior) and its damping (which favors stabilization of Hb levels) with competing effects, obscuring its net impact on the Hb dynamics. This reflects the difficulties in isolating and adjusting any single treatment-related parameter (such as ESA half-life) such that both Hb targets are reached quickly and cycling is mitigated at the same time.

#### Onset of self-sustained cycling

Self-sustained hemoglobin cycling corresponds to a periodic solution (a limit cycle) of the system Eq (12). A Hopf bifurcation marking the onset of such a limit cycle occurs when the steady state, Eq (13), becomes unstable via a sign change of the real part of two complex conjugate roots of *χ*(*α*). The approximation for *α*_±_ given by Eq (23) goes through such a sign change when the composite parameter *γ* goes through zero from above (i.e., when ‘damping’ turns into ‘forcing’ in the above harmonic oscillator analogy), suggesting that *γ* is an approximation for the control parameter governing the Hopf bifurcation. Hence, the condition *γ* < 0 translates into an approximate condition for the presence of cycles in the smoothed model. Restoring the original parameter dependence, using the definitions given by Eq (5), this yields the condition

$$\begin{array}{c} \frac{\mu^{\prime }c_{0}}{\sigma \tau_{\text{int}}^{2}}≳\frac{\text{ln}\;2}{\tau T_{\text{ESA}}T_{\text{RBC}}}\;, \end{array}$$ (25)

see gray arrow in Fig 4A. Among others, this approximation suggests that close to the onset of self-sustained cycling, higher ESA responsiveness (*μ*), a steeper dosing chart with larger dose changes (*c*_0_), larger physiological delays (e.g., comprising cell cycle times of the erythropoietic lineage) (*τ*) and a larger ESA half-life (*T*_ESA_) may promote cycling while a larger width of the Hb target window (*σ*) and larger intervals between ESA doses (*τ*_int_) inhibit cycling. A higher ESA responsiveness and a narrow Hb target range have indeed been suggested previously as contributors to Hb cycling [5].

#### Transient cycling after perturbations

Perturbations of the system (such as bleedings) can induce transient cycling even in regimes in which self-sustained cycling is not possible (Fig 7). This is the case if the system parameters are located sufficiently close to the self-sustained cycling regime. Such transient oscillations decay over time with a characteristic time scale that becomes the longer the closer the system is to the onset of self-sustained permanent oscillations. Formally, this is reflected by the fact that the system Eq (12) exhibits damped cycles in the vicinity of the steady state when the roots of *χ* acquire an imaginary part. Hence, the approximation Eq (23) implies that transient damped cycles occur if $\gamma^{2}≲4\beta m$, where *β*, *γ* and *m* are defined in Eqs (20) and (22).

## Supporting information

S1 File Model simulation codes. Python codes needed to run model simulations and plot figures.
(ZIP)
